# Can Inflammation-Resolution Provide Clues to Treat Patients According to Their Plaque Phenotype?

**DOI:** 10.3389/fphar.2019.00205

**Published:** 2019-03-07

**Authors:** Gabrielle Fredman

**Affiliations:** Department of Molecular and Cellular Physiology, Albany Medical College, Albany, NY, United States

**Keywords:** inflammation, resolution, resolvin, atherosclerosis, efferocytosis

## Abstract

Inflammation-resolution is an active process that is governed in part by specialized pro-resolving mediators (SPMs) such as lipoxins, resolvins, protectins, and maresins. SPMs, which are endogenously biosynthesized, quell inflammation and repair tissue damage in a manner that does not compromise host defense. Importantly, failed inflammation-resolution is an important driving force in the progression of several prevalent diseases including atherosclerosis. Atherosclerosis is a leading cause of death worldwide and uncovering mechanisms that underpin defective inflammation-resolution and whether SPMs themselves can revert the progression of the disease are of utmost clinical interest. Because atherosclerosis is a disease in which low-grade persistent inflammation results in tissue injury, SPMs have garnered immense interest as a potential treatment strategy. This mini review will highlight recent work that describes mechanisms associated with defective inflammation-resolution in atherosclerosis, as well as the protective actions of SPMs and their potential use as a therapeutic.

## Introduction

Atherosclerotic cardiovascular disease (CVD) is a chronic inflammatory disease that is characterized by the accumulation of lipids and cells in the vessel wall. Inflammation increases atherothrombosis, which can manifest itself as result of ruptured or eroded plaques (Libby et al., 2014). Patients are typically treated with lipid lowering drugs like statins and in some cases with PCSK9 inhibitors. Importantly, some patients on lipid lowering drugs still succumb to acute atherothrombotic events and have a “residual risk” due to the patient’s poorly controlled inflammation (Ridker, 2016). Ridker et al. (2017) recently published a landmark study in which the initial results from the Canakinumab Anti-inflammatory Thrombosis Outcome Study (CANTOS) were uncovered. Canakinumab is a human monoclonal antibody that targets interleukin-1β, a major pro-inflammatory cytokine associated with CVD. Importantly, Canakinumab significantly decreased re-occurring CVD events, independent of lipid lowering (Ridker et al., 2017). These results are the first to show that an anti-inflammatory agent can provide clinical benefit in CVD patients and are therefore a considerable advance to the field. An adverse effect of the drug was fatal infection, which suggests that blocking pro-inflammatory ligands may suppress critical host defense mechanisms (Tabas and Glass, 2013). In this regard, this trial also opens the field for further investigation to find new mechanisms and targets that temper exuberant immune responses without increased risk for infection.

## Can You Limit Inflammation and Spare Host Defense?

Acute inflammation is a process that protects us against microbial invaders (Kumar et al., 2005). However, when acute inflammation becomes excessive or persistent, chronic inflammation occurs which can lead to tissue damage (Kumar et al., 2005). Previously it was thought that acute inflammation terminated by passive means. But we now appreciate that the termination of acute inflammation is an active process that involves the biosynthesis of specific chemical mediators called specialized pro-resolving mediators (SPMs) (Serhan, 2014). SPMs are endogenously generated and are derived from the omega-6 fatty acid arachidonic acid (AA) and from omega-3 fatty acids including eicosapentaenoic acid (EPA) and docosahexaenoic acid (DHA). They comprise four major families called lipoxins, resolvins of the E- and D-series, protectins and maresins (Serhan, 2014). SPMs actively counter balance pro-inflammatory signals and stimulate tissue reparative/regenerative programs (Serhan, 2014) and are the body’s natural brakes to inflammation. Most importantly, SPMs are not immunosuppressive (Spite et al., 2009; Chiang et al., 2012). Therefore they may be an ideal therapeutic strategy to treat progressive diseases, like atherosclerosis.

## Inflammation-Resolution Is Defective in Atherosclerosis

The balance between SPMs and pro-inflammatory mediators (like leukotriene B_4_, LTB_4_) during acute inflammation regulates the swift onset of inflammation-resolution (Chiang et al., 2012; Fredman et al., 2012, 2014). Moreover, an imbalance between SPMs and pro-inflammatory mediators has been linked to several chronic inflammatory diseases in humans, including, COPD, asthma (Basil and Levy, 2016), aggressive periodontitis (Freire and Van Dyke, 2013), arthritis, and atherosclerosis (Brezinski et al., 1992; Fredman et al., 2016). In this regard, it is no surprise that administration of SPMs is protective in several disease models including sepsis, asthma, periodontitis, arthritis, injury-induced neointimal hyperplasia, myocardial infarction, stroke and atherosclerosis (Merched et al., 2008; Hasturk et al., 2015; Fredman et al., 2016; Salic et al., 2016; Viola et al., 2016).

Specialized pro-resolving mediators act on distinct cellular targets and evoke specific and generalized functions in tissues. Some examples of their general functions are to promote the clearance of apoptotic cells, (i.e., efferocytosis), quell pro-inflammatory factors and decrease excessive oxidative stress (OS) (Chiang et al., 2012; Fredman et al., 2012). Notably an example of distinct actions of SPMs is on human platelets in which the SPM Resolvin E1 (RvE1) potently inhibits ADP-induced platelet aggregation, whereas Protectin D1 (PD1) does not (Dona et al., 2008). These protective features of SPMs become particularly important in the context of atherosclerosis because efferocytosis is defective (Tabas, 2010; Kojima et al., 2016), pro-inflammatory signals and OS are unchecked, which results in necrosis and tissue damage. Therefore, SPMs may be ideal to reduce several of the maladaptive processes associated with atherosclerosis in a manner that does not compromise host defense. This mini review will highlight recent literature that demonstrates a protective role for SPMs in atherosclerosis.

### Specialized Pro-resolving Mediators in Atherosclerosis

Serhan et al. (2003) uncovered that angioplasty increased intracoronary levels of lipoxins in humans, which was one of the first insights into the role of SPMs in atherosclerosis (Brezinski et al., 1992). Animal studies were then investigated to study a causative role for SPM in atherosclerosis. Overexpression of a key biosynthetic enzyme for SPMs (Shen et al., 1996; Serhan et al., 2003) called 15-lipoxygenase (15-LOX), in rabbits or transgenic 12/15-LOX-*ApoE^-/-^* mice led to a significant decrease in atherosclerosis (Shen et al., 1996; Serhan et al., 2003). Moreover, peritoneal macrophages isolated from 12/15-LOX-*ApoE^-/-^* transgenic mice exhibited an increased production of key SPMs, compared with wild type control macrophages (Merched et al., 2008). In this study, the 12/15-LOX-*ApoE^-/-^* transgenic mice were fed a chow (i.e., low cholesterol, low saturated fat) diet. Importantly, 15-LOX (or 12/15-LOX), can also lead to the pro-atherogenic oxidation of LDL (Cyrus et al., 1999; Serhan et al., 2003; Merched et al., 2008; Poeckel and Funk, 2010; Merched et al., 2011). The composition of the diet is an important determinant of whether these pathways promote SPM production or pro-atherogenic lipids. As an example, when the 12/15-LOX-*ApoE^-/-^* transgenic mice were fed a Western Diet (i.e., high cholesterol, high saturated fat), atherosclerosis progression was exacerbated compared with *ApoE^-/-^* controls (Merched et al., 2011). These studies suggest that diet may be critical for the formation of protective pro-resolving ligands. More recent studies focused on the roles of SPMs as therapeutics.

The first study that demonstrated a therapeutic role for SPMs in atherosclerosis was by Hasturk et al. (2015). This study uncovered that RvE1, when treated orally and topically (i.e., to the gums) thwarted atherogenesis in a rabbit model of atherosclerosis (Hasturk et al., 2015). Moreover, RvE1 also decreased atherogenesis in the context of periodontal disease, which is a known risk factor for atherosclerosis in humans (Van Dyke and Starr, 2013). The oral-topical application suggests a new therapeutic administration strategy for SPMs and is plausible for long-term treatment (Hasturk et al., 2015). Mechanisms that underpin RvE1’s actions in this context remain underexplored, but may be linked to RvE1’s ability to reduce leukocyte infiltration into inflamed tissues (Dona et al., 2008). A subsequent study revealed that RvE1 was protective during atheroprogression in mice as well (Salic et al., 2016). In this regard, Salic et al. uncovered that RvE1 and atorvastatin reduced lesion size significantly more than RvE1 alone (Salic et al., 2016). The mechanisms underlying the additional benefit of the co-treatment are unknown. One thought is that statins in other contexts, boost 15-epi-LXA_4_ (or ATL) and a new class of SPMs called 13-series resolvins (or RvTs) (Birnbaum et al., 2006; Dalli et al., 2015), and so the presence of statins may further enhance endogenous SPM production to promote protection. Further studies need to be conducted to determine if RvTs were generated in that context. Together, these two studies suggest that RvE1 is protective in atherosclerosis models.

Importantly RvE1 is derived from the omega-3 fatty acid eicosapentaenoic acid (EPA). RvE1’s known protective actions on atherogenesis, atheroprogression and on platelets (via infra) is of particular interest (Dona et al., 2008; Fredman et al., 2010), given the new results from the REDUCE-IT study that tested the actions of EPA on cardiovascular outcomes. This study involved over 8,000 patients who had elevated triglyceride levels and who were at elevated cardiovascular risk (i.e., had a previous cardiovascular event or diabetes with one additional risk factor). The results revealed the EPA group had a ∼25% relative risk reduction in the first occurrence of a major adverse events which were defined as cardiovascular death, non-fatal myocardial infarction, non-fatal stroke, unstable angina requiring hospitalization, or coronary revascularization (Bhatt et al., 2017, 2019). The mechanism for protection is not known and one possibility is that EPA drives the formation of E-series SPM, like RvE1. Another potential clue into mechanism was that all of the patients were on statins. As mentioned above, RvT biosynthesis is enhanced in the presence of atorvastatin (Dalli et al., 2015; Walker et al., 2017) and it is possible that RvTs or E-series SPM are increased in these patients. The REDUCE-IT study did not measure E-series resolvins or RvTs so more studies are needed to determine the role SPMs in this context.

D-series SPMs, which are compounds that are derived from the omega-3 fatty acid docosahexaenoic acid (DHA) were identified in human atherosclerotic plaques (Fredman et al., 2016). Specially, RvD1 was defective in regions of human atherosclerotic plaques that were highly necrotic, had exuberant OS and thin caps, compared with regions that had less necrosis, reduced OS and thicker fibrous caps (Fredman et al., 2016). Not only was RvD1 decreased, but there was also a marked imbalance between RvD1 and LTB_4_ in these highly necrotic plaque regions (Fredman et al., 2016). A subsequent study observed a correlation between intima medial thickness and the RvD1:LTB_4_ ratio measured in salivary samples (Thul et al., 2017), suggesting that this ratio may be a predictor of atherosclerosis in humans. To test mechanism and causation, less necrotic (i.e., early plaques) and highly necrotic plaques (i.e., advanced plaques) from *Ldlr^-/-^* were monitored for SPMs, LTs and other eicosanoids (Fredman et al., 2016). RvD1 was significantly reduced in advanced murine plaques compared with early, less necrotic plaques, which was similar to the human plaque findings (Fredman et al., 2016). Importantly when RvD1 was administered to mice with established atherosclerosis, the intraplaque RvD1:LTB_4_ ratio was restored to that of early less-necrotic plaques. Also, RvD1 treatment led to a decrease in lesional necrosis and oxidative stress and increased fibrous cap thickness (Fredman et al., 2016), which are features that are characteristic of “stable” plaques. A parallel study in *Apoe^-/-^* mice revealed that Resolvin D2 (RvD2) and Maresin 1 (Mar1) were decreased as atherosclerosis progressed (Viola et al., 2016). Importantly, restoration of RvD2 and Mar1 promoted features of plaque stability as well (Viola et al., 2016). Lastly, ATL also promoted features of plaque stability in mice (Petri et al., 2017). Therefore, this new burst of literature suggests that a common feature of SPMs in advanced murine atherosclerosis is to enhance repair (i.e., decrease necrosis and increase collagen) of plaques. The protective actions of pro-resolving proteins and peptide, like AnnexinA1 and Ac2-26 in atherosclerosis have also been reported and are mentioned in the following review (Heinz et al., 2017). Generally, AnnexinA1 or Ac2-26 limit atherogenesis and plaque necrosis (Drechsler et al., 2015; Fredman et al., 2015; Kusters et al., 2015), which is similar to the actions of SPMs. Not surprisingly, some of the SPM that were mentioned above (e.g., RvD1 and ATL) bind the same receptors as AnnexinA1 and Ac2-26, which may account for the similar actions in plaques. The major gaps in the field now are a deeper mechanistic understanding of SPM biosynthesis and signaling in plaques.

A clue into mechanism associated with defective biosynthesis of SPM in plaques was recently uncovered by the Tabas lab and involves the efferocytosis receptor on macrophages called MerTK (Cai et al., 2016). It is known that efferocytosis promotes the biosynthesis of SPMs (Schwab et al., 2007; Norling et al., 2012; Fredman et al., 2014) and SPM administered to macrophages enhances in efferocytosis (Godson et al., 2000; Serhan, 2014), suggesting an important feed-forward pro-resolution circuit for an efficient clearance response. Therefore it is no surprise that efferocytosis is a major cellular program of the inflammation-resolution response and mechanisms associated with defective efferocytosis may inform our knowledge as to why SPM may not be efficiently synthesized. Under conditions of inflammation or OS, MerTK undergoes an ADAM17 mediated cleavage event, which renders the receptor inactive and results in a soluble fraction called soluble Mer (sol-Mer) (Sather et al., 2007; Thorp et al., 2011). A role for MerTK cleavage in human plaques has recently emerged in which histological staining of plaque tissue sections revealed that macrophages near necrotic cores had lower MerTK and higher ADAM17 (Garbin et al., 2013). More recently, increased sol-Mer was observed in human symptomatic plaques compared with asymptomatic lesions, again suggesting a role for MerTK cleavage (Cai et al., 2017). Soluble Mer increased in *Ldlr*^-/-^ mice as atherosclerosis progressed to the advanced stage as well (Cai et al., 2017). To test whether MerTK cleavage itself was important in the formation of the necrotic core, the Tabas lab generated mice that resist MerTK cleavage (termed cleavage resistant or *Mertk^CR^* mice) (Cai et al., 2016) and transferred myeloid cells from *WT* or *Mertk^CR^* into WD-fed *Ldlr^-/-^* mice. The plaques from the *Mertk^CR^* mice had increased lesional SPMs, a higher SPM:LTB_4_ ratio, increased efferocytosis, thicker fibrous caps, and decreased lesional necrosis compared with controls (Cai et al., 2017). The increase of SPMs may act in a feed-forward circuit because RvD1 was shown to limit MerTK cleavage on macrophages *in vitro* and administration of RvD1 to *Ldlr^-/-^* mice resulted increased lesional MerTK (i.e., less MerTK cleavage), compared with controls (Fredman et al., 2016; Cai et al., 2017). These data suggest another feed-forward pro-resolution circuit in which MerTK signaling promotes SPM biosynthesis, which in turns prevents MerTK cleavage. Therefore, MerTK plays a critical role in regulating both efferocytosis and SPM biosynthesis. Other studies uncovered that the cyclin dependent kinase inhibitor 2b or anti-CD47 antibody treatment reduced atheroprogression by promoting efferocytosis in mice (Kojima et al., 2014, 2016). Whether SPMs were generated by these treatment strategies is not known and knowledge gained from these studies would be of immense clinical interest.

### Rupture and Erosion Prone Plaques: Examples of Failed Inflammation-Resolution

Vulnerable plaques in humans are a type of atherosclerotic plaque that are at risk for precipitating acute atherothrombotic clinical events, including stroke and myocardial infarction (Virmani et al., 2006). Rupture of the thin cap can lead to a cascade of events that culminate in thrombotic events. Moreover, these plaques have distinct features, including large areas of necrosis, a thin layer of collagen that overlies the areas of necrosis, heightened pro-inflammatory factors and increased OS (Schrijvers et al., 2005; Virmani et al., 2006; Tabas, 2010). As mentioned above, SPMs tempered pro-inflammatory factors, quelled lesional OS, decreased necrotic cores and promoted a more stable-like plaque phenotype (Fredman et al., 2016; Viola et al., 2016; Petri et al., 2017). Among of the most striking and consistent observations in the mice treated with SPMs was an increase in fibrous cap thickness and/or collagen synthesis in plaques (Fredman et al., 2015, 2016; Viola et al., 2016; Petri et al., 2017). SPM-initiated mechanism(s) that drive the formation of the protective cap remain unknown. A plausible hypothesis that efferocytosis (which is known to promote TGFβ release), or macrophage phenotype switching to a less proteolytic state can participate in the repair process. Along these lines, SPMs also enhance the phagocytosis of blood clots *in vitro*, suggesting that SPM may play a role in clot remodeling and other tissue repair programs (Elajami et al., 2016).

While thin caps and large necrotic cores underpin a subset of plaques that are prone to rupture, there is also a phenotypically distinct plaque that is known to cause atherothrombotic events. These plaques exhibit features of superficial endothelial cell (EC) erosion and are almost completely opposite to the characteristics of the above plaques because they have an abundance of extracellular matrix (notably proteoglycans and glycosaminoglycans), harbor fewer macrophages and inflammatory cells and they lack large lipid pools (Kolodgie et al., 2002). Interestingly, these plaques are associated with the female sex, smoking, younger age, hypertriglyceridemia and diabetes (Libby and Pasterkamp, 2015). Multiple mechanisms may contribute to EC erosion, including TLR signaling and neutrophil trafficking an/or a modification to the subendothelial matrix that may trigger loss of adhesion or apoptosis of ECs (Quillard et al., 2017).

### Mechanisms for Plaque Erosion and a Potential Role for SPMs in Preventing This Process

Histologically, eroded plaques exhibit those of “stable” thick-cap fibroatheromas and have minimal markers of inflammation (Campbell et al., 2014). However, as we begin to learn more about the mechanisms that underpin this process, inflammation, yet again comes to the forefront. Briefly, disruption of ECs contribute to acute thrombotic complications that result from eroded plaques (Quillard et al., 2017). EC activation and apoptosis drives EC denudation and thrombus formation (Quillard et al., 2017). An interesting mechanistic clue was found in plasma from patients with eroded plaques in which myeloperoxidase (MPO) an enzyme primarily found in neutrophils (PMN) was markedly increased, compared with those of ruptured lesions (Ferrante et al., 2010). Moreover, other experiments revealed that PMN, when co-cultured with ECs, led to EC injury and the formation of hypochlorous acid (a major product of MPO) to trigger EC apoptosis (Sugiyama et al., 2004; Villanueva et al., 2011). Therefore, PMN and their ability to generate harmful products from MPO including other factors like reactive oxygen species (ROS) are thought to be involved in plaque erosion. Another mechanism involves the PMN’s ability to undergo NETosis under certain inflammatory conditions. NETosis is a form of cell death in which nuclear chromatin is released into the extracellular space. This process likely evolved to ensnare bacteria and heal wounds through the activation of coagulation pathways (Martinod and Wagner, 2014). However, NETosis and the formation NETs (neutrophil extracellular traps) are maladptive in atherosclerosis (Doring et al., 2017) and Quillard T. et al. described that NETs from PMN are associated with superficial erosion (Quillard et al., 2015). More recent studies followed up on this observation in which NETs were directly shown to activate ECs (Folco et al., 2018). The activation of TLR2, an innate immune system pattern recognition receptor, has also been implicated in plaque erosion (Quillard et al., 2015). In fact TLR2 agonists promoted a low-level activation and dysfunction of ECs, which lead to the expression of E-selectin, VCAM-1, endoplasmic reticulum stress OS and ultimately apoptosis (Quillard et al., 2015). Together, plaque erosion presents a major issue and methods to identify and treat these types of plaques are limited.

#### SPMs Limits Excessive PMN Recruitment, Limit MPO Activity and Decrease NET Formation and Quells EC Activation

There are several clues in the literature that suggest that SPMs may be ideal molecules to prevent or control the many factors that contribute to EC erosion. As mentioned above, activated neutrophils are thought to be major contributors to EC erosion. Interestingly, SPMs are known to limit excessive recruitment of PMN to tissues in several contexts including ischemia reperfusion injury, local inflammation driven by infectious or sterile stimuli and acute lung injury (Arita et al., 2007; El Kebir et al., 2009). Given the importance of MPO in plaque erosion, it is salient to note that ATL has been shown to reduce MPO-mediated pro-inflammatory circuits (El Kebir et al., 2009). Moreover, Lipoxin A_4_ (LXA_4_) and the pro-resolving peptide Ac2-26 were shown to limit NET formation (Tibrewal et al., 2014). Together, SPM act to control PMN trafficking as well as NET formation and may play a protective role on PMN that interact with atherosclerotic ECs.

Specialized pro-resolving mediator also exert protective actions on ECs. Earlier results indicated that LXA_4_ stimulated prostacyclin (PGI_2_) from human ECs and blocked EC-PMN interactions human ECs, suggesting a role for LXs is hemostasis and vascular inflammation (Brezinski et al., 1989; Papayianni et al., 1996). Another more recent study found that RvD1 maintains EC integrity by preventing LPS mediated barrier functions (Zhang et al., 2013; Chattopadhyay et al., 2017). Together these studies highlight how SPM act on ECs to potentially prevent several features that drive superficial erosion.

#### SPMs Decrease Platelet Activation and Thrombosis

The clinical manifestations of atherothrombosis are myocardial infarction and stroke (Foley and Conway, 2016). There are several reports in the literature that suggest a protective role for SPMs in thwarting thrombosis. Earlier studies revealed that healthy humans receiving low-dose aspirin were able to generate ATL (Chiang et al., 2004, 2006). Importantly, ATL was inversely correlated with pro-thrombotic thromboxane (Chiang et al., 2004, 2006). As mentioned above, RvE1, which can also be generated in the presence of low-dose aspirin, blocks ADP and thromboxane-stimulated platelet aggregation (Dona et al., 2008; Fredman et al., 2010). Moreover, mechanistic studies revealed that RvE1, via its receptor called ERV1 blocked ADP-induced signals downstream of the P2Y_12_ receptor (Fredman et al., 2010). More recently, RvD2 prevented thrombosis and necrosis of the dermal vascular network in a mouse burn model (Bohr et al., 2013).

Platelets and their interactions with leukocytes are also critical for both inflammation and a swift resolution response. On the one hand, transient platelet:PMN aggregates can be protective because the can promote SPM biosynthesis (Abdulnour et al., 2014; Norris et al., 2018). In this context, SPMs also reduce human platelet:PMN aggregates, which suggest an important temporal regulation between the formation and dissociation of these aggregates for swift resolution response (Abdulnour et al., 2014; Norris et al., 2018). On the other hand, platelet:PMN aggregates are also known to contribute to plaque inflammation (Gerdes et al., 2016). Therefore, these results suggest that pathologic platelet-leukocyte aggregates may be defective in their ability to generate SPMs. It would be interesting to determine the types and/or expression levels of the integrins and other surface receptors involved in the protective versus pathologic aggregate formation. In this regard, the addition Mar1 promotes a pro-resolving phenotype of platelets and prevents thrombin-activated platelets from releasing soluble CD40L, CD62P, thromboxane, and platelet microparticles (Lannan et al., 2017). Together, results from the above studies collectively demonstrate that SPM play important roles in regulating platelet activation, which could have important therapeutic implications for CVD.

### New Therapies to Treat CVD

Unlike traditional anti-inflammatory strategies that in many cases lead to immunosuppression, pro-resolving mediators actively counteract inflammation without compromising host-defense (Serhan, 2014). In fact, SPMs lower the threshold for antibiotic therapy and enhance host defense mechanisms with regard to viral and bacterial infections and (Chiang et al., 2012; Morita et al., 2013). Because atherosclerosis is a long-tem progressive disease in which blocking inflammation may lead to unintended host defense issues, a promising strategy would be to promote inflammation-resolution in CVD patients to combat chronic inflammation as well as promote tissue repair.

In the future, it will be important to determine more detailed signaling mechanisms associated with SPM, as well as if SPM can be effectively targeted atherosclerotic plaques. Along these lines, microparticles released from activated immune cells carry SPMs (Norling et al., 2011). This raises the possibility that nanoparticles derived from activated human leukocytes could potentially be vehicles for targeted delivery (Norling et al., 2011). Another possibility is plaque-targeted polymeric nanoparticles that contain pro-resolving ligands (Fredman et al., 2015). Also, delivery of SPMs through biodegradable vascular wraps limited restenosis and local inflammation (Lance et al., 2017; Wu et al., 2017) and may also be an important strategy for targeted delivery of SPM.

In addition to the possibility of targeted SPM therapeutics, another important aspect to consider is the time of day for optimal treatment. Heart attacks typically occur in the morning (Thosar et al., 2018) and a new link between circadian rhythms and inflammation-resolution has been uncovered. In fact, recent work demonstrated an important role for circadian rhythm and the biosynthesis of SPM (Colas et al., 2018). Briefly, Colas R. et al. observed that a particular class of SPMs were regulated in a diurnal manner and that patients with CVD exhibited a disturbed regulation of these mediators (Colas et al., 2018). Therefore, timing of treatment may be particularly important for successful outcomes.

Moreover, SPM comprise a family of numerous mediators. SPMs each have distinct structures and activate specific GPCRs, which suggest that they have both generalized and distinct actions. An important parallel in the immune system are chemokines that comprise several distinct members and activate specific GPCRs. Not surprisingly, decades of work revealed that chemokines have both generalized and specific actions. Generalized functions of SPMs and chemokines highlight the evolutionary importance of these pathways. Nevertheless, inflammation-resolution is an emerging field and a deeper understanding of how SPMs signal will provide insight into which SPMs or combination of SPMs may be ideal to treat diseases, like atherosclerosis.

## Author Contributions

The author confirms being the sole contributor of this work and has approved it for publication.

## Conflict of Interest Statement

The author declares that the research was conducted in the absence of any commercial or financial relationships that could be construed as a potential conflict of interest.
